# No evidence for a reciprocal relationship between daily self-control failures and addictive behavior in a longitudinal study

**DOI:** 10.3389/fpsyg.2024.1382483

**Published:** 2024-05-01

**Authors:** Anja Kräplin, Mohsen Joshanloo, Max Wolff, Juliane Hilde Fröhner, Christian Baeuchl, Klaus-Martin Krönke, Gerhard Bühringer, Michael N. Smolka, Thomas Goschke

**Affiliations:** ^1^Department of Psychology, Technische Universität Dresden, Dresden, Germany; ^2^Department of Psychology, Keimyung University, Daegu, Republic of Korea; ^3^Department of Psychiatry and Psychotherapy, Technische Universität Dresden, Dresden, Germany; ^4^Mind Foundation, Berlin, Germany; ^5^Department of Psychiatry and Psychotherapy, Charité—Universitätsmedizin Berlin, Berlin, Germany; ^6^Institut für Therapieforschung, Munich, Germany

**Keywords:** self-control, addictive behaviors, ecological momentary assessment, longitudinal study, cross-lagged panel

## Abstract

**Introduction:**

We all experience occasional self-control failures (SCFs) in our daily lives, where we enact behaviors that stand in conflict with our superordinate or long-term goals. Based on the assumption that SCFs share common underlying mechanisms with addictive disorders, we tested the hypothesis that a generally higher susceptibility to daily SCFs predicts more addictive behavior, or vice versa.

**Methods:**

At baseline, 338 individuals (19–27 years, 59% female) from a community sample participated in multi-component assessments. These included among others (1) a clinical interview on addictive behaviors (quantity of use, frequency of use, DSM-5 criteria; *n* = 338) and (2) ecological momentary assessment of SCFs (*n* = 329, 97%). At the 3-year and 6 year follow-up, participation rates for both assessment parts were 71% (*n* = 240) and 50% (*n* = 170), respectively.

**Results:**

Controlling for age, gender, IQ, and baseline addiction level, random-intercept cross-lagged panel models revealed that participants who reported more SCFs also showed pronounced addictive behavior at the between-person level, but we found no evidence of a predictive relationship at the within-person level over time.

**Discussion:**

A higher rate of SCFs is associated with more addictive behavior, while there is no evidence of an intraindividual predictive relationship. Novel hypotheses suggested by additional exploratory results are that (1) only addiction-related SCFs in daily life are early markers of an escalation of use and thus for addictive disorders and that (2) an explicit monitoring of SCFs increases self-reflection and thereby promotes the mobilization of cognitive control in response to goal-desire conflicts.

## 1 Introduction

Among mental disorders, addiction is the most obvious and common example of impaired self-control (Heatherton and Wagner, 2011; Kelley et al., 2015). A progressive loss of control, despite the person’s awareness of the serious negative consequences, and despite the person’s persistent desire to stop the behavior, are key diagnostic criteria of addictive disorders (American Psychiatric Association [APA], 2013). In this paper, we focus on substance-related (i.e., alcohol and tobacco use) and non-substance-related (i.e., gaming, gambling, shopping, internet use) addictive behaviors. As one of the first studies, we report longitudinal, cross-lagged data to examine whether a generally higher proneness to daily SCFs predicts more addictive behaviors or vice versa.

Self-control can be defined as the ability to regulate one’s behavior, thoughts, and emotions according to desired goals and values despite conflicting immediate desires (de Ridder et al., 2012). Researchers often equate low self-control with impulsivity because self-control involves the successful regulation of impulses (de Ridder et al., 2012). Based on trait models of impulsivity and on integrative models of self-control (Kotabe and Hofmann, 2015; Milyavskaya et al., 2019; Inzlicht et al., 2021; Goschke and Job, 2023), we assume that trait impulsivity is one of several factors that may predispose individuals toward an increased proneness to commit self-control failures. However, self-controlled behavior (or a lack thereof) depends on additional factors that are not covered by the term impulsivity in a narrow sense (e.g., whether individuals use precommitment strategies to avoid temptations; Crockett et al., 2013; Kotabe and Hofmann, 2015; Studer et al., 2019) or whether they develop beneficial habits (Galla and Duckworth, 2015; Duckworth et al., 2018) may lead to far-sighted choices without requiring interventive self-control strategies and impulse control.

Self-control is considered to be relevant in various behavioral domains and serves to regulate a wide range of desire types such as eating, sleeping or smoking (Hofmann et al., 2012b). Theoretical models and the corresponding empirical evidence have shown that self-control is based on various cognitive functions and underlying brain mechanisms such as inhibitory control, attention control, the modulation of value-based decision-making, performance monitoring, as well as preventive strategies such as a precommitment (Fujita, 2011; de Ridder et al., 2012; Krönke et al., 2018, 2020a; Inzlicht et al., 2021; Overmeyer et al., 2021; Goschke and Job, 2023). These mechanisms have also been considered important for the development and course of addictive behavior (Goschke, 2014; Tang et al., 2015; Brand et al., 2019; Ceceli et al., 2022). Within the scope of this paper, addictive behavior is broadly defined, using quantity and frequency of use (Rehm et al., 2013) as well as the level of addictive disorder severity (American Psychiatric Association [APA], 2013). Research over the past decades has shown that individuals with addictive behavior have impairments in the underlying components of self-control and functional changes in associated brain networks compared to healthy controls such as impaired inhibitory control (for reviews, see Luijten et al., 2014; Smith et al., 2014), altered value-based decision-making (for review, see Amlung et al., 2017), and impaired performance monitoring (for review see, Luijten et al., 2014).

While impaired self-control emerges as an important characteristic of addictive behavior, the predictive relationship between general self-control in daily life (across different desire types) and addictive behavior in particular remains an important open research question. We hypothesized that a general impairment of self-control in daily life should predict a higher proneness to addictive behavior as a specific domain of SCFs. Using ecological momentary assessments (EMA), we and other research groups have shown that people frequently experience conflict-laden desires in their daily lives (e.g., to eat unhealthy food, check social media during work, etc.), which often result in SCFs, i.e., failures to resist these desires in order to pursue long-term goals (Hofmann et al., 2012a; Wolff et al., 2016, 2020; Overmeyer et al., 2021). Given that failures in everyday self-control are common, and assuming that addictive behaviors are partly mediated by impairments of self-control, individuals with a generally greater proneness to SCFs should also have a higher risk of developing addictive behavior. Thus, our first hypothesis is that a generally higher rate of SCFs across different domains of everyday life is predictive of more future addictive behaviors (Hypothesis 1).

Complementary to this assumption, addictive behaviors are known to have detrimental effects on cognitive control, causing alterations in brain networks involved in reward, learning, and executive functions (Meruelo et al., 2017; Lees et al., 2020), which are important neurocognitive mechanisms underlying self-control in daily life. Even in addictive behaviors without substance use and its neurotoxic effects on the brain, such as Internet Gaming Disorder, changes in reward processing may occur as a result of conditioning in the often immediately rewarding environment of internet gaming (Kräplin et al., 2021). As a result of these changes in the neurocognitive mechanisms underlying self-control, both addiction-related (e.g., drinking a glass of beer instead of the planned soft drink) and non-addiction-related SCFs (e.g., texting on WhatsApp instead of writing the planned paper) may increase over time. In summary, SCFs may be both a predisposing risk factor for the onset of addictive behavior and may worsen over time as the disorder develops. Thus, our second hypothesis is that more addictive behavior predicts more SCFs in daily life (Hypothesis 2).

Based on this theoretical background, in the present study, we applied a cross-lagged panel model with three time points to analyze the hypothesized causal processes using longitudinal data. With the three time points, it is possible to include a random intercept in the cross-lagged panel models, which accounts for trait-like, time-invariant stability (RI-CLPM; Hamaker et al., 2015). This random intercept partials out the between-person variance, so that the lagged relationships in the RI-CLPM are actually related to within-person dynamics. Due to the non-experimental nature of observational studies, it is also important to consider covariates to facilitate causal interpretations. Based on the existing literature on addictive behavior and its development over time in young adults (Mortensen et al., 2006; Wittchen et al., 2008; Sjölund et al., 2015), we assumed that older age, male gender and lower intelligence would be associated with higher baseline scores and greater increases in addictive behavior over time. We therefore included these covariates in our longitudinal analyses.

The innovative aspect of this paper is that our data allows us to investigate the reciprocal relationship between daily SCFs and addictive behaviors within a cross-lagged panel model. The findings may thus shed new light on the etiology and early markers, as well as the possible consequences of addictive behavior in daily life, and thus inform prevention and intervention strategies.

## 2 Materials and methods

We report how we determined our sample size, all data exclusions, all manipulations, and all measures in this study part. Data was collected as part of the prospective longitudinal community study “Volitional dysfunction in self-control failures and addictive behaviors” within the Collaborative Research Centre SFB 940 “Volition and Cognitive Control” at theTUD Dresden University of Technology, Germany (study protocol at ClinicalTrial.gov NCT04498988 and on the OSF at https://osf.io/yu5rm/).

### 2.1 Design and procedure

This longitudinal cohort study started in 2012 and will end in 2024. Participant recruitment began in 2013 and included seven overlapping waves of data collection, with each wave lasting three years. Participants were invited to three multi-component assessments (at baseline and after 3 and 6 years) and additionally to annual clinical assessments only (at 1, 2, 4, 5 and 7 years). The multi-component assessment at baseline, 3-year and 6-year follow-up consisted of (1) clinical assessments and questionnaires (e.g., trait impulsivity) in an interview room (at baseline at local PCs) and later by telephone (with online questionnaires), (2) ecological momentary assessments (EMA) of SCFs in daily life, (3) an experimental task battery in the laboratory (assessing cognitive control abilities and decision-making), and (4) structural and functional neuroimaging using magnetic resonance imaging (MRI). Annual follow-ups were scheduled according to the date of the last of the four baseline sessions, i.e., the fMRI session. The 1-, 2-, 4-, 5-, and 7-year follow-ups included only the clinical assessment via telephone interviews. In line with the scope of this paper, only measures from the clinical assessments and the EMA at baseline, and at the 3- and 6-year follow-ups are described in detail below. Previous publications based on data from our sample focused either on the prediction of SCFs by neurocognitive characteristics (Wolff et al., 2016, 2020; Krönke et al., 2020a,b, 2021) or on the prediction of addictive behaviors by neurocognitive characteristics (Kräplin et al., 2020, 2022). Please refer to these studies and our OSF registration^1^ for detailed information on hypotheses, samples, and materials.

### 2.2 Recruitment and participants

Between 2013 and 2016, random samples of 18,000 inhabitants aged between 19 and 27 from Dresden, Germany, were invited by post to participate in the study of whom 10.3% responded (see Supplementary material for sample size calculation). At baseline, we included participants who met the criteria for one of the following three groups (for sample characteristics, see Table 1):

**TABLE 1:** Demographic characteristics of the baseline sample with means (M) and standard deviations (SD) or numbers (n) and percentages for the substance-related disorder (SUD) group, the behavioral addiction (BA) group, and the control group.

Baseline sample	SUD	BA	Control
*N* = 338	100	118	120
	**M (SD)**	**M (SD)**	**M (SD)**
Age	21.8 (1.6)	21.8 (1.7)	21.9 (1.8)
Intelligence quotient	103.7 (8.9)	104.4 (10.1)	104.8 (10.4)
	***n* (%)**	***n* (%)**	***n* (%)**
Female participants	53 (53.0%)	70 (59.3%)	76 (63.3%)
Income ≤ 1,000 Euro per month	75 (75.8%)	92 (77.0%)	89 (75.4%)
School graduation gymnasium^a^^,^^b^	70 (70.7%)	87 (73.7%)	98 (83.0%)
In education, pupils, or students	72 (72.7%)	87 (73.7%)	87 (73.7%)^b^

^a^Gymnasium is a type of secondary schools existing in Germany, which qualifies for university entrance.

^b^Three participants had missing values.

(1) Substance use disorder (SUD) group: in the last 12 months, participants met two or more criteria for an alcohol and/or tobacco use disorder according to the fifth edition of the Diagnostic and Statistical Manual of Mental Disorders (DSM-5; American Psychiatric Association [APA], 2013), but had no lifetime behavioral addiction.
(2) Behavioral addiction (BA) group: in the last 12 months, participants met two or more criteria for a DSM-5 gambling disorder or adapted, unofficial criteria for addictive disorders related to gaming, shopping and/or internet use (not related to gambling, gaming or shopping). The adapted criteria were based on the DSM-5 SUD criteria, as there were no official criteria for these disorders at the start of recruitment. To achieve a homogeneous group definition, we defined the BA group as individuals who met at least two of the 11 adapted SUD criteria. Participants in the BA group had no lifetime SUD.
(3) Control group: participants had no lifetime BA or SUD diagnoses.

We used data from the baseline assessments of the three groups to test cross-sectional hypotheses with a sample including a sufficient number of participants with high levels of addictive behaviors and addictive disorders for the longitudinal analyses. Please note that for the longitudinal analyses reported here the groups are not relevant for the to-be-tested hypotheses. However, group membership is included as a control variable in our longitudinal analyses.

**FIGURE 1:**
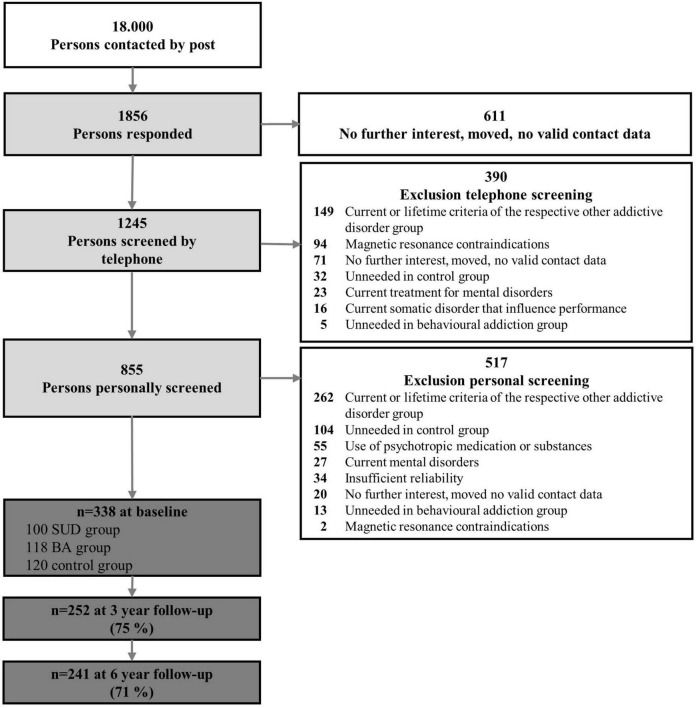
Participant flowchart with numbers and reasons for inclusion and exclusion. SUD, substance use disorder; BA, behavioral addiction.

Exclusion criteria for all participants were (1) a limited ability to provide written informed consent or to understand the questionnaires and tasks, (2) disorders that might influence cognition or motor performance, (3) magnetic resonance contraindications, (4) current treatment for mental disorders, or (5) use of psychotropic medications or substances. After applying the inclusion and exclusion criteria, 855 participants were invited to a face-to-face diagnostic screening. In the personal screening, we used the Munich-Composite International Diagnostic Interview (DIA-X/M-CIDI, Wittchen and Pfister, 1997) to assess the following exclusion criteria: (6) lifetime psychotic symptoms, bipolar disorder, and other SUDs or BAs not under study, and (7) major depression, somatoform, anxiety, obsessive-compulsive, or eating disorders in the last 4 weeks. At the end of the recruitment phase, first the control group and then the behavioral addiction group were filled. All other recruited persons who would have been eligible for these groups were then excluded, as they were no longer needed. In the end, 338 participants were included in the study (Figure 1).

### 2.3 Measurements

#### 2.3.1 Addictive behaviors

An important aim of our study was to use comparable measures for all forms of addictive behavior. We therefore defined addictive disorders uniformly based on SUDs (see “2.2 Recruitment and participants”) and decided to also use the quantity and frequency of use for non-substance-related addictive behaviors, which are also important indicators for substance-related ones (Rehm et al., 2013). According to our own previous work (Kräplin et al., 2020, 2022) and our pre-registration^2^, addictive behavior was operationalized by three measures including consumption (quantity, frequency) and addictive disorder severity based on a combination of all expressions of addictive behavior (i.e., alcohol use, tobacco use, internet use, gaming, gambling, shopping):

(1) Quantity of use: The amount of use on a typical occasion (e.g., a typical drinking occasion) was assessed for each of the addictive behaviors (grams of ethanol for drinking, number of cigarettes for smoking, and hours of use for internet use, gaming, gambling, and shopping). The different values for quantity of use were normalized (i.e., rescaled to range between 0 and 1) to have only positive values for the later addition. Normalization was carried out in a long data format to make the values comparable across the different addictive behaviors and time points (baseline and follow-ups), and then summed up to form a composite score at each time point. The sum can range between 0 and 6, according to the rescaling from 0 to 1 and the 6 addictive behaviors.
(2) Frequency of use: The frequency of use was assessed as days per week for each of the addictive behaviors. The different frequencies of use were summed up across the different addictive behaviors into a composite score at each time point. The sum can range between 0 and 42, according to the maximum of seven days per week and the 6 addictive behaviors.
(3) Addictive disorder severity: The DSM-5 criteria were assessed for each of the addictive behaviors. For substance and gambling-related disorders, DSM-5 criteria were available at the baseline assessment of the study. For disorders related to gaming, shopping, or internet use (that was not related to gambling, gaming, or shopping), we applied adapted criteria and questions from the DSM-5 SUD (e.g., “Have you ever tried unsuccessfully to limit the use of the internet for a few days?”). All fulfilled addictive disorder criteria were summed up to a score at each time point. The sum can range between 0 and 64, according to the maximum of 11 DSM-5 criteria (with the exception of gambling disorder with 9 criteria) and the 6 addictive disorders.

Quantity of use, frequency of use, and addictive disorder severity were all self-reported in clinical interviews conducted in person or by telephone. The standardized diagnostic interviews were conducted by advanced psychology students (i.e., Master students in clinical psychology with a Bachelor’s degree in psychology) trained in clinical assessment and supervised by the first author. Interviewers were instructed to ensure that the reported consumption behavior and the diagnostic criteria were separated between the different addictive behaviors. All 338 participants were assessed at baseline, 252 at the 3-year follow-up (75% of the original sample), and 241 (71%) at the 6-year follow-up.

#### 2.3.2 Self-control failures

We used an established EMA procedure from our working group (Wolff et al., 2016, 2020), which was based on a procedure by Hofmann et al. (2012a). Over seven days, we assessed the occurrence of desires, the desire type selected from a list of 19 categories (based on Hofmann et al., 2012b), the desire intensity, whether these desires were conflict-laden, the intensity of the conflict, whether participants tried to resist the desire and whether they enacted the desire (Table 2). For this purpose, participants were provided with study smartphones running only the EMA application movisens XS (version 1.3.3; movisens GmbH, Karlsruhe, Germany). Participants received eight alarms per day, which were randomly issued within a 14-h time window starting at 8, 9, or 10 AM, depending on the participant’s preference. When an alarm was accepted, participants were asked to complete a short questionnaire on the device to assess the occurrence of SCFs in the hour prior to the alarm. Depending on the response rate, each participant completed up to 56 questionnaires. SCFs were operationalized as the enactment of conflict-laden desires divided by the number of questionnaires in which a conflict was reported (percentage of SCFs ranging from 0 to 1). At baseline, 331 participants were assessed with EMA (*n* = 7 missing due to technical difficulties); at 3-year follow-up, 240 (71% of the original sample); and at 6-year follow-up, 170 (50%). Fewer people participated in EMA than in the clinical assessment because they were more willing to answer questions over the phone than to come to the lab to retrieve the study smartphone for EMA.

**TABLE 2:** Experience sampling questionnaire (according to Wolff et al., 2020).

Item	Wording	Response format	Condition for presenting item
1. Desire	Was there at some point during the last 60 min a situation where you had a desire and an opportunity to enact a certain behavior?	Yes/No	Alarm accepted
2. Domain of desire	Which of the following categories^a^ fits the desire most?	19 options^a^	“Yes” response to Item 1
3. Desire strength	How strong was the desire on a scale from 1 (very weak) to 6 (very strong)?	Likert scale (1 to 6)	“Yes” response to Item 1
4. Conflict	In this situation, were you aware of any reason why you should not enact the desire?	Yes/No	“Yes” response to Item 1
5. Conflict strength	How strong was your conviction that you should not enact the desire on a scale from 1 (very weak) to 6 (very strong)?	Likert scale (1 to 6)	“Yes” response to Item 4
6. Resistance	Did you attempt to resist the desire?	Yes/No	“Yes” response to Item 4
7. Enactment	Did you (at least in part) enact the desire?	Yes/No	“Yes” response to Item 1

The original questionnaire was written in German language.

^a^Eating; drinking (no alcohol); drinking (alcohol); smoking; taking other substance; using the internet; playing a computer game; watching TV; buying something; gambling; exercising; sleeping; resting; retreating; misbehaving; socializing; having sex or intimacy; using the bathroom; other.

#### 2.3.3 Covariates

As in our previous work (Kräplin et al., 2020, 2022) and as explained in the introduction, we used the covariates age, gender, and IQ at baseline. Moreover, the group allocation at baseline is, by definition, related to addictive use and addictive disorder severity and was therefore included as a covariate. The measurement of group membership has already been explained above. Age and gender were assessed at the first face-to-face appointment using a modified version of the DIA-X/M-CIDI (Wittchen and Pfister, 1997). Intelligence quotients (IQ) were assessed at the second appointment using the Hamburg-Wechsler Adult Intelligence Scale-Revised (HAWIE; Tewes, 1994).

### 2.4 Statistical analysis

#### 2.4.1 Confirmatory analyses

To test the competing hypotheses of predictive relationships between SCFs and addictive behavior^2^, we conducted random intercept cross-lagged panel models (RI-CLPM; Hamaker et al., 2015) within a structural equation modeling (SEM) framework. The RI-CLPM includes a random intercept to account for “time-invariant, trait-like stability” (Hamaker et al., 2015 p. 104). The random intercept corresponds to the stable trait variance. The inclusion of this stable trait component changes the meaning of the autoregressive part of the model. Whereas in the CLPM the cross-lagged paths reflect the associations between variables over time, in the RI-CLPM these paths reflect the associations between wave-specific deviations from an individual’s stable trait level. This allows for the separation of associations on the between- and within-person level and the interpretation of the cross-lagged associations as causal effects (Hamaker et al., 2015; Usami, 2021).

All models were estimated with observed variables and robust maximum likelihood (MLR) using Mplus 8.10 (Muthén and Muthén, 1998–2017). The data and analysis scripts can be found on OSF^3^. Estimations were done under missing data theory, using all available data. Data was analyzed using full information maximum likelihood (FIML) to handle missing data. For the EMA, seven cases were missing at baseline due to technical difficulties. In addition, the assessment of some participants took slightly longer than the planned seven days. In this case, we identified the first full sampling day with eight alarms, counted seven days from that point, and deleted all further responses outside this period. Regarding the data on addictive behavior, we have not excluded any data as all were plausible. In addition to our pre-registration, we applied a log transformation to the frequency of use variable as the distribution was non-normal.

**FIGURE 2:**
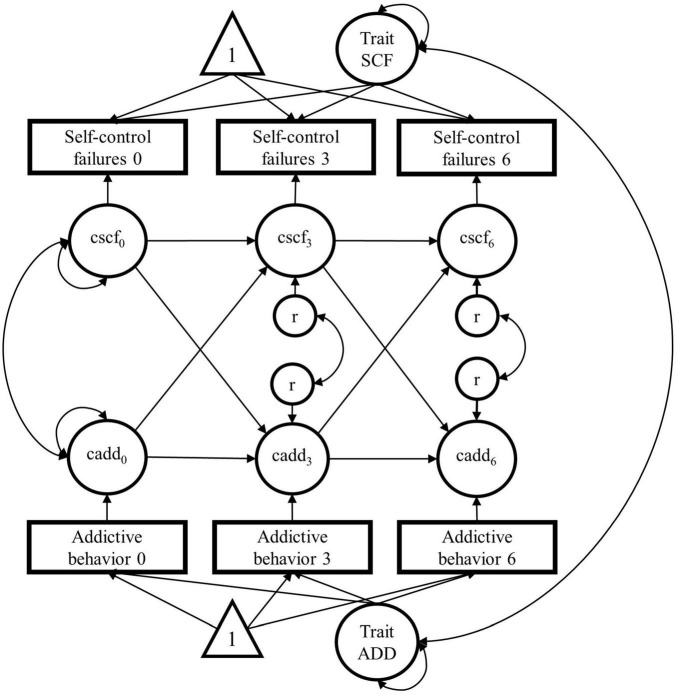
Proposed random intercept cross-lagged panel model (RI-CLPM; Hamaker et al., 2015) for the causal relationship between daily self-control failures and addictive behavior over three time points (baseline, 3-year and 6-year follow-ups). Triangles represent constants (for the mean structure), squares represent observed variables, and circles represent latent variables. SCF, self-control-failures; ADD, addictive behavior (we will specify 3 models for each indicator of addictive behavior: quantity of use, frequency of use, and DSM-5 criteria); cscf/cadd, within-person centered variables; r, residual variance of the within-person-centered variables; time points are indicated by the numbers: 0 = baseline, 3 = 3-years follow-up, 6 = 6-years follow-up.

In the RI-CLPM, we assessed the temporal stability (auto-regressive effects) over time and the temporal ordering of the variables (cross-lagged effects) between SCFs and addictive behavior. The proposed model is shown in Figure 2. The Mplus input can be found in the OSF document^2^. We specified three models for each indicator of addictive behavior (quantity of use, frequency of use, DSM-5 criteria). In sum, we ran three RI-CLPMs without covariates.

In the next step, we added the covariates age, gender, IQ, and group membership at baseline by regressing all observed variables (at times 0, 3, and 6) on these four variables. We used the models with covariates for inference. The Mplus input can be found in the OSF document^2^. In sum, we ran three RI-CLPMs with covariates.

We used 95% confidence intervals and the standard *p* < 0.05 criteria to determine whether our coefficients are significantly different from zero. Model fit was evaluated using several fit indices. For the Chi-square statistic, a small, non-significant value indicates a good fit. As this parameter should be used with caution (e.g., the null hypothesis is a perfect fit, and it is affected by large sample sizes), alternative incremental fit indices were also used for the evaluation of the model fit: the comparative fit index (CFI), the root mean square error of approximation (RMSEA), and the standardized root mean square residual (SRMR). For the Chi-square statistic, a small, non-significant value indicates a good fit. For the CFI, values greater than 0.95 indicate a good fit, and values between 0.90 and 0.95 indicate an acceptable fit. RMSEA and SRMR values smaller than 0.08 indicate good fit (Kline, 2015).

#### 2.4.2 Exploratory analyses

##### 2.4.2.1 Self-control failures related to addictive behavior

Regarding the desire domains in our SCF questionnaire, one could argue that we are making circular inferences because the reported SCFs also include some desire domains related to addictive behavior (e.g., drinking alcohol) and we want to predict addictive behavior from the SCFs in these desire situations. Therefore, we separated SCFs based on the respective desire domain. The rationale for separating the desire domains was based on the addictive behavior on which our study focused. We computed SCFs separately for desire domains not related to addictive behavior (e.g., sleeping, watching TV, for the full list see Table 2) and desire domains related to those addictive behaviors of interest in our study (drinking alcohol, smoking, gaming, shopping, and gambling) and tested whether our hypotheses are still valid. As SCFs in desire domains related to addictive behavior were partly very lowly correlated with each other (*r* = −0.06 for SCFs at Baseline and at FU6), there was no need for a random intercept to capture between-person variance. Therefore, we constrained the variance of the random intercept for this variable to zero and removed the covariance between the two intercepts for SCFs and the addictive behavior. With this change, we acknowledge that 100% of the variance of SCFs related to addictive behavior is at the within-person level.

##### 2.4.2.2 Motivational vs. volitional self-control failures

**FIGURE 3:**
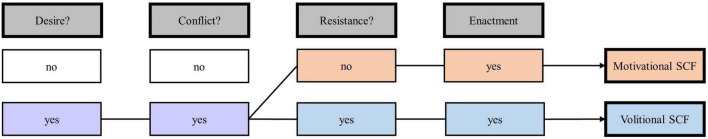
Definition of motivational self-control failures (SCFs) and volitional SCFs (adapted from Hofmann and Kotabe, 2012) according to the answers in the experience sampling questionnaire applied in this study (Wolff et al., 2020).

Additionally to our definition of SCFs above, we explored whether our results differ depending on the definition of SCFs either as volitional or motivational SCFs (Hofmann and Kotabe, 2012). We defined volitional SCFs as SCFs, for which participants indicated that they had attempted to resist a conflicting desire, and motivational SCFs as SCFs in which participants indicated that they had not attempted to resist the conflicting desire (see Figure 3).

### 2.5 Ethics

The study procedures were carried out in accordance with the Declaration of Helsinki. The Institutional Review Board (IRB00001473) of the TUD Dresden University of Technology approved the study protocol under the reference EK45022012. Before beginning the initial online survey, all participants were informed about the study and all participants provided informed consent.

## 3 Results

### 3.1 Descriptive data

Descriptive data on the SCFs are shown in Table 3. Participants responded to approximately 80% of the 56 issued alarms (8 alarms per day) during one week [Baseline (BL): 43 (77%); 3-year follow-up (FU3): 44 (79%); 6-year follow-up (FU6): 45 (80%)]. Of the about 30 desires reported (BL: 32%; FU3: 31%; FU6: 32%), about 35% were conflict-laden (BL: 36%; FU-3: 35%; FU6: 35%) and 19% were SCFs (BL: 19%; FU3: 19%; FU6: 19%). This also means that about half of the participants reported SCFs in situations in which they perceived a conflict between a current desire and a long-term goal (Table 3). Interindividual variability was large, ranging from no SCFs to reports of SCFs in all conflict-laden situations. The proportion of desire types reported was similarly distributed in the three waves. The most frequently reported desire types of the 19 types in total were eating (BL: 24%; FU3: 30%; FU6: 29% across all participants and situations) followed by drinking (no alcohol) (BL: 8%; FU3: 9%; FU6: 10%), using the bathroom (BL: 8%; FU3: 9%; FU6: 10%), relaxing (BL: 7%; FU3: 7%; FU6: 8%), and sleeping (BL: 7%; FU3: 7%; FU6: 6%).

**TABLE 3:** Descriptive data of self-control failures (SCFs), quantity of use, frequency of use, and number of fulfilled DSM-5 criteria.

	*N*	M	SD	Range
SCF0 (%)	321	53	25	0–100
SCF3 (%)	228	53	27	0–100
SCF6 (%)	165	55	27	0–100
Quant0	338	0.37	0.26	0–1.6
Quant3	253	0.74	0.28	0–2.5
Quant6	242	0.79	0.25	0.1–1.6
Frequ0	338	6.71	4.44	0.25–21.3
Frequ3	253	5.64	4.21	0–20
Frequ6	242	5.34	4.10	0.25–21.5
DSM0	338	3.03	2.73	0–16
DSM3	253	2.14	2.69	0–16
DSM6	242	2.03	2.47	0–14

SCF = Enactments of conflict-laden desires divided by the number of questionnaires in which a conflict was indicated (percent of SCFs range from zero to 100). Quant = Quantity of use on a typical occasion assessed for each of the addictive behaviors (gram ethanol, cigarettes, and hours). The different values for quantity of use were normalized (i.e., rescaled to range between 0 and 1) and then summed up to form a composite score at each time point. The sum can range between 0 and 6, according to the rescaling from 0 to 1 and the 6 addictive behaviors. Frequ = Frequency of use assessed as days per week for each of the addictive behaviors. The different frequencies of use were summed up over the different addictive behaviors into a composite score at each time point. The sum can range between 0 and 42, according to the maximum of seven days per week and the 6 addictive behaviors. DSM = (adapted) DSM-5 criteria were assessed for each of the addictive behaviors. All fulfilled addictive disorder criteria were summed up to one score at each time point. The sum can range between 0 and 64, according to the maximum of 11 DSM-5 criteria (with the exception of gambling disorder with 9 criteria) and the 6 addictive disorders.

The descriptive data on addictive behavior are also presented in Table 3. However, the values of the combined measures are difficult to interpret. Therefore, we have included the detailed descriptive data for all three waves and each addictive behavior (i.e., alcohol use, tobacco use, internet use, gaming, gambling, and shopping) in Supplementary Table 1 in Supplementary material.

The correlation matrix between SCFs and addictive behavior (quantity of use, frequency of use, and DSM-5 criteria for addictive disorders) is presented in Supplementary Tables 2-1–2-3 in Supplementary material. The correlation coefficients are mostly small to moderate and positive. As an interest emerged during the review process, we also explored the correlation between the SCFs and trait impulsivity, a construct that may predispose individuals to a higher proneness of SCFs (Supplementary Table 3). Trait impulsivity was measured using the Barratt Impulsiveness Scale (BIS-11; Patton et al., 1995; German version: Preuss et al., 2003). In sum, impulsivity and SCFs are significantly but weakly (*r* < 0.18) correlated.

### 3.2 Confirmatory analyses

#### 3.2.1 Quantity of use and self-control failures

The model without covariates yielded an acceptable model fit (χ^2^ = 17.29, df = 5, *p* = 0.004, RMSEA = 0.085 [0.044–0.131], CFI = 0.901, SRMR = 0.064) and the model with covariates yielded a good model fit (χ^2^ = 14.485, df = 11, *p* = 0.207, RMSEA = 0.031 [0.000–0.069], CFI = 0.988, SRMR = 0.031). This supports the interpretation of the model with covariates, as we had planned in the pre-registration.

The parameter estimates of the model with covariates are presented in Table 4 (for the models without covariates, see Supplementary Table 4-1). The autoregressive coefficients were significant for the quantity of use, which was also found in the model without covariates. This suggests that there is stability in the quantity of use beyond the trait-like stability captured by the random intercept. As shown in Table 4, none of the cross-lagged paths was significant in the hypothesized direction (also in the model without covariates, Supplementary Table 4-1). At the between-person level, the relationship between the random intercept factors was significantly positive but small. More SCFs are associated with more addictive behavior, but the between-person correlations do not capture within-person variation over time and therefore do not indicate directionality. In summary, there is no evidence to support the hypothesis that higher levels of addictive behavior predict more daily SCFs or vice versa.

**TABLE 4:** Parameter estimates for the random intercept cross-lagged panel models (RI-CLPM; Hamaker et al., 2015) including self-control failures (SCFs) and quantity of addictive behaviors.

				Confidence interval		
**Predictor**	**Outcome**	**Unstandardized coefficient**	***p***	**Lower**	**Upper**	**Standardized coefficient**
**Autoregressive paths**						
SCFs 0 → SCFs 3		−0.10	0.52	−0.39	0.20	−0.10
SCFs 3 → SCFs 6		−0.10	0.52	−0.39	0.20	−0.09
Quantity 0 → Quantity 3		0.21	0.01	0.05	0.36	0.18
Quantity 3 → Quantity 6		0.21	0.01	0.05	0.36	0.21
**Cross-lagged paths**						
SCFs 0 → Quantity 3		−0.10	0.24	−0.26	0.07	−0.09
SCFs 3 → Quantity 6		−0.10	0.24	−0.26	0.07	−0.09
Quantity 0 → SCFs 3		−0.21	0.04	−0.40	−0.01	−0.20
Quantity 3 → SCFs 6		−0.21	0.04	−0.40	−0.01	−0.22
**Covariance (between)**						
Trait SCFs ↔ Trait quantity		0.01	0.02	0.001	0.02	0.70

Equality constraints have been added to autoregressive and cross-lagged paths. The model includes the covariates age, gender, IQ, and group membership at baseline. The parameter estimates for the model without covariates can be found in Supplementary Table 4-1 in Supplementary material.

#### 3.2.2 Frequency of use and self-control failures

The model without covariates yielded a poor model fit (χ^2^ = 21.591, df = 5, *p* = 0.001, RMSEA = 0.099 [0.059–0.144], CFI = 0.862, SRMR = 0.052) and the model with covariates yielded a good model fit (χ^2^ = 17.572, df = 11, *p* = 0.09, RMSEA = 0.042 [0.000–0.077], CFI = 0.978, SRMR = 0.026). This again speaks in favor of interpreting the model with covariates.

As shown in Table 5, none of the autoregressive coefficients were significant (also in the model without covariates, Supplementary Table 4-2). This suggests that there is not a strong stability in the frequency of use and SCFs beyond the trait-like stability captured by the random intercept. In addition, none of the cross-lagged paths was significant in the hypothesized direction (also in the model without covariates, Supplementary Table 4-2). At the between-person level, the relationship between the random intercept factors was significantly positive and small. More SCFs are related to a higher frequency of addictive behavior on the between-person level. In sum, there is no evidence to support the hypothesis that a higher frequency of addictive behaviors predicts more daily SCFs or vice versa.

**TABLE 5:** Parameter estimates for the random intercept cross-lagged panel models (RI-CLPM; Hamaker et al., 2015) including self-control failures (SCFs) and frequency of addictive behaviors.

				Confidence interval		
**Predictor**	**Outcome**	**Unstandardized coefficient**	***p***	**Lower**	**Upper**	**Standardized coefficient**
**Autoregressive paths**						
SCFs 0 → SCFs 3		−0.13	0.33	−0.40	0.14	−0.14
SCFs 3 → SCFs 6		−0.13	0.33	−0.40	0.14	−0.12
Frequency 0 → Frequency 3		−0.01	0.90	−0.14	0.12	−0.02
Frequency 3 → Frequency 6		−0.01	0.90	−0.14	0.12	−0.004
**Cross-lagged paths**						
SCFs 0 → Frequency 3		−0.21	0.07	−0.43	0.01	−0.23
SCFs 3 → Frequency 6		−0.21	0.07	−0.43	0.01	−0.12
Frequency 0 → SCFs 3		−0.16	0.00	−0.26	−0.06	−0.29
Frequency 3 → SCFs 6		−0.16	0.00	−0.26	−0.06	−0.14
**Covariance (between)**						
Trait SCFs ↔ Trait frequency		0.01	0.02	0.002	0.02	0.45

Equality constraints have been added to autoregressive and cross-lagged paths. The model includes the covariates age, gender, IQ, and group membership at baseline. In addition to our pre-registration, we applied a log transformation to the frequency of use variable as the distribution was non-normal. The parameter estimates for the model without covariates can be found in Supplementary Table 4-2 in Supplementary material.

#### 3.2.3 DSM-5 criteria and self-control failures

The model without covariates yielded a good fit (χ^2^ = 14.016, df = 5, *p* = 0.016, RMSEA = 0.073 [0.029–0.120], CFI = 0.951, SRMR = 0.042) and also the model with covariates (χ^2^ = 8.610, df = 5, *p* = 0.126 RMSEA = 0.046 [0.000–0.097], CFI = 0.991, SRMR = 0.019). We again will interpret the model with covariates as preregistered.

As shown in Table 6, the autoregressive coefficients for the DSM-5 criteria were significant, which was not the case in the model without covariates (Supplementary Table 4-3). This suggests that there is stability in the DSM-5 criteria beyond the trait-like stability captured by the random intercept when controlling for age, gender, IQ, and group membership at baseline. None of the cross-lagged paths were significant in the hypothesized direction (also in the model without covariates, Supplementary Table 4-3). At the between-person level, the association between the random intercept factors was significantly positive and small. In conclusion, there is no evidence that more fulfilled DSM-5 criteria predict more daily SCFs or vice versa.

**TABLE 6:** Parameter estimates for the random intercept cross-lagged panel models (RI-CLPM; Hamaker et al., 2015) including self-control failures (SCFs) and DSM-5 criteria of addictive disorders.

				Confidence interval		
**Predictor**	**Outcome**	**Unstandardized coefficient**	***p***	**Lower**	**Upper**	**Standardized coefficient**
**Autoregressive paths**						
SCFs 0 → SCFs 3		−0.07	0.64	−0.36	0.22	−0.07
SCFs 3 → SCFs 6		−0.07	0.64	−0.36	0.22	−0.07
Criteria 0 → Criteria 3		0.31	0.002	0.11	0.50	0.21
Criteria 3 → Criteria 6		0.31	0.002	0.11	0.50	0.37
**Cross-lagged paths**						
SCFs 0 → Criteria 3		−1.55	0.06	−3.15	0.05	−0.13
SCFs 3 → Criteria 6		−1.55	0.06	−3.15	0.05	−0.16
Criteria 0 → SCFs 3		−0.02	0.09	−0.04	0.003	−0.15
Criteria 3 → SCFs 6		−0.02	0.09	−0.04	0.003	−0.21
**Covariance (between)**						
Trait SCFs ↔ Trait criteria		0.10	0.001	0.04	0.16	0.60

Equality constraints have been added to autoregressive and cross-lagged paths. The model includes the covariates age, gender, IQ, and group membership at baseline. The parameter estimates for the model without covariates can be found in Supplementary Table 4-3 in Supplementary material.

### 3.3 Exploratory analyses

We further explored the effect of two variables on our results found in the confirmatory RI-CLPMs: (1) the desire type (addictive vs. non-addictive) and (2) the self-control failure type (volitional SCFs vs. motivational SCFs).

Concerning the desire type, we ran separate RI-CLPMs for desire domains related to addictive behaviors of interest in our study [= (potentially) addictive desire types, e.g., drinking alcohol, computer gaming] and desire domains not related to addictive behavior (= non-addictive desire types, e.g., sleeping, watching TV). For (potentially) addictive desire types, we found a significantly positive cross-lagged path from more SCFs to a later higher quantity of use (see Supplementary Table 5 in Supplementary material). For frequency of use and DSM-5 criteria, these paths were not significant. For non-addictive desire types, significant negative cross-lagged paths were found, i.e., higher quantity and frequency was associated with later lower non-addictive SCFs (see Supplementary Tables 6, 7 in Supplementary material). This direction is in line with the results of our confirmatory analyses. There was also a significant cross-lagged path between higher non-addictive SCFs and later lower quantity of use. For the DSM-5 criteria, the models did not differ between the two types of desire domains and from the confirmatory results. In sum, these analyses shed new light on the confirmatory results. First, the hypothesized positive association between SCFs and later addictive behavior may only be true for (potentially) addictive SCFs. Second, the negative, counterintuitive association between higher SCFs and later less addictive behavior seems to hold only for non-addictive SCFs. It seems important to separate the two types of SCFs. Importantly, non-addictive SCFs do not seem to be the first step to addictive behavior.

Concerning the type of SCFs, we ran separate RI-CLPMs for volitional SCFs (which involved attempts to resist conflicting desires) and motivational SCFs (involving no attempts to resist conflicting desires). The negative, counterintuitive association between higher SCFs and later lower addictive behavior appears to hold only for motivational SCFs. A higher rate of motivational SCFs significantly predicts later lower frequency of use and lower DSM-5 criteria (see Supplementary Tables 8, 9 in Supplementary material). Conversely, a higher frequency of use predicts fewer future motivational SCFs. Due to the resulting insufficient number of SCFs, it was not possible to combine both exploratory questions and, for example, analyze only motivational non-addictive SCFs. Overall, we found exploratory evidence that motivational SCFs, where a person does not even try to resist a desire despite knowing that this desire conflicts with a long-term goal, predict less addictive behavior in the future and vice versa. To understand this counterintuitive relationship and to relate it to the first exploratory analysis (desire type), we further explored the reported conflict strength and the desire types of these motivational SCFs. Participants who reported situations with motivational SCFs reported significantly lower conflict strength compared to situations with volitional SCFs (BL: *M_*mot*_* = 2.90, *M*_*vol*_ = 3.67, *t* = 11.15, *p* < 0.01; FU3: *M*_*mot*_ = 2.78, *M*_*vol*_ = 3.53, *t* = 9.61, *p* < 0.01; FU6: *M_*mot*_* = 2.80, *M_*vol*_* = 3.68, *t* = 8.96, *p* < 0.01). Participants who reported situations with motivational SCFs also reported significantly more (potentially) addictive desires in these situations compared to situations with volitional SCFs, especially more smoking- and alcohol-related desires over all three time points and gaming-related desires at the 3-year and the 6-year follow-up (see Supplementary Table 10 in Supplementary material). In sum, motivational SCFs were reported as less conflict-laden and were more often reported in situations with (potentially) addictive desire types.

## 4 Discussion

Failure to resist immediate desires and temptations that stand in conflict with the pursuit of superordinate or long-term goals (SCFs) is common in people’s daily lives (Hofmann et al., 2012a; Wolff et al., 2016). To our knowledge, this study is the first to use longitudinal data to investigate whether addictive behavior predicts an escalation in daily SCFs, or whether more daily SCFs in turn predict an increase in addictive behavior. Confirmatory cross-lagged panel analyses provided evidence for neither of these reciprocal hypotheses. Additional exploratory analyses provided ideas for interpretation and further hypothesis testing.

By combining EMA and clinical assessments of addictive behavior in a cross-lagged panel design over 6 years, we tested whether SCFs in different domains of daily life lead to more addictive behavior and/or whether the amount of addictive behaviors predicts in turn the rate of daily SCFs. SCFs were defined as situations in which individuals act on a desire that conflicts with their long-term goals. Addictive behavior was operationalized via the quantity and frequency of use and the degree to which DSM-5 criteria for addictive disorders were met. We applied a random intercept cross-lagged panel models to control for between-person differences, so that cross-lagged relationships relate only to within-person variance over time. In our confirmatory analyses, we found evidence for a positive association between the rate of SCFs and the amount of addictive behavior at the between-person level. This is in line with the assumption that compared to other mental disorders, addiction in particular is strongly associated with repeated failures of self-control (Bühringer et al., 2008; Goschke, 2014; Kozak et al., 2019). Additional exploratory analyses have shown that trait impulsivity may constitute a predisposition that increases the proneness to commit daily SCFs, which has previously been shown to be a good predictor of addictive behavior in relation to alcohol use, tobacco use (Granö et al., 2004) or gambling (Dowling et al., 2017). However, as we had hypothesized on the basis of integrative models of self-control (Kotabe and Hofmann, 2015; Milyavskaya et al., 2019; Inzlicht et al., 2021; Goschke and Job, 2023), the low correlation also indicates that SCFs are driven by additional mechanisms not directly related to trait impulsivity, as, for instance, dysfunctional performance monitoring (Krönke et al., 2018; Overmeyer et al., 2021).

The innovative feature of our study is that we additionally tested the association between SCFs and addictive behavior at the within-person level, i.e., within individuals over time. However, at the within-person level, we found no evidence in support of the hypothesis that more SCFs predict more addictive behavior or vice versa. Although these null findings should be cautiously interpreted until replicated in future within-person studies, our additional exploratory analyses offer several potential explanations.

In our exploratory analyses, we further analyzed the effects of two variables on our results: (1) the desire type (addictive vs. non-addictive) and (2) the SCF type (volitional SCFs vs. motivational SCFs). Concerning the desire type, we found evidence for the hypothesized positive relationship between SCFs and addictive behavior only for SCFs elicited by (potentially) addictive behaviors, whereas a negative relationship was found for SCFs involving non-addictive behaviors. This indicates that the desire type is important for the interpretation of our results. These exploratory findings on the relationship between SCFs and addictive behavior can be interpreted in the light of choice models of self-control (Inzlicht et al., 2021). In these models, self-control is conceived of as the result of a valuation process in which different response options are assigned subjective values. A decision about which option to act upon is made through a dynamic integration of these competing values into a common value signal (Berkman et al., 2017). This fits with our process model of self-control, according to which SCFs depend on whether representations of long-term consequences are activated at the moment of choice and exert an impact on the computation of an integrated value signal encoded in the ventromedial prefrontal cortex (Hare et al., 2009; Krönke et al., 2020a). Consistent with this assumption, there is evidence that individuals showing an insufficient impact of long-term outcomes on this neural value signal show a higher proneness to commit daily SCFs (Krönke et al., 2020a). Moreover, recent findings suggest that purely cognitive knowledge of long-term consequence is not sufficient to support far-sighted decisions, but anticipated long-term consequences (e.g., the prospect of bad health) promote self-control especially if they evoke emotional responses at the time of choice (and thereby presumably modulate the neural value signal in the ventromedial prefrontal cortex that ultimately determines choice) (Kruschwitz et al., 2019; Walter et al., 2020). Importantly, a deficient monitoring network that leads to insufficient mobilization of cognitive control (Inzlicht et al., 2015; Kotabe and Hofmann, 2015) presumably leads to a reduced impact of anticipated future outcomes on this integrated value signal in the ventromedial prefrontal cortex, increasing the weight of short-term rewards on the value signal and the likelihood of self-control failures (Hare et al., 2009; Goschke, 2014; Krönke et al., 2020a). Inadequate performance monitoring may thus be a risk factor for addictive behaviors because the value signal is then more strongly determined by the short-term rewards, whereas long-term rewards (e.g., good health) are not sufficiently integrated into the value signal, even though individuals may be aware of these long-term consequences on a purely cognitive level.

In conclusion, SCFs involving (potentially) addictive desires may be early markers of addictive behavior or even addictive disorders. It is particularly interesting that we found that more non-addictive SCFs predict less addictive behavior and vice versa. One possible albeit speculative explanation could be that non-addictive SCFs lead to more self-reflection and mobilization of cognitive control in general, which may also lead to lower rates of later addictive behavior. This speculation is in line with empirical evidence (Krönke et al., 2018; Overmeyer et al., 2021) suggesting that less self-controlled behavior could be explained by reduced mobilization of cognitive control as a consequence of insufficient performance monitoring. The reporting of SCFs in our study may have acted as a form of intervention that supports the monitoring of SCFs and thereby strengthens cognitive control.

A second result of our exploratory analyses was that an inverse relationship between the rate of SCFs and addictive behavior was evident for motivational SCFs, for which individuals reported that they had not even tried to resist desires that conflicted with their goals. Interestingly, the desires in situations with motivational SCFs were more often (potentially) addiction-related and the level of conflict strength was lower compared to volitional SCFs. This can be interpreted in line with our speculative assumption that the daily assessment of SCFs with the EMA may have strengthened monitoring processes. The monitoring of such motivational self-control failures in addiction-related desire types could lead to an increase in the previously too-low conflict strength with superordinate or long-term goals (Inzlicht et al., 2014) and a stronger mobilization of cognitive control (Inzlicht et al., 2015; Kotabe and Hofmann, 2015). Higher conflict strength and more mobilized cognitive control in the next addiction-related conflict situation could in turn lead to greater success in resisting the addiction-related desire. All these speculative explanations are based on our exploratory analyses and need to be tested in a confirmatory approach in future studies.

### 4.1 Strengths and limitations

A strength of our study is that we were able to recruit a large representative community sample of adults aged 19 to 27. The young age cohort allows for analyses of early developmental processes that may lead to addictive behavior. Although the recruitment was representative, the registration for participation in the study could be subject to selection biases (e.g., more likely to be young and female, higher school education, for details, see Kräplin et al., 2022). By design, there was also a high baseline proportion of individuals with only mild and at best moderate addictive disorder severity in the addiction groups. These biased sample characteristics (younger, more females, higher school education, and lower addictive disorder severity) would lead to an underestimation of the true associations between SCFs and addictive behavior. Concerning methods, in each of the three assessment waves we conducted an intensive EMA for one week with eight prompts per day to assess volitional and motivational SCFs. This has the advantage of providing an ecologically valid assessment of SCFs. A disadvantage is that this intensive reporting may also lead to monitoring processes that affect future SCFs. However, this cannot be avoided with the EMA method. In addition, we did not capture preventive (anticipatory) self-control (e.g., Studer et al., 2019) and beneficial habits (e.g., Galla and Duckworth, 2015), which have been shown to have an important influence on self-controlled behavior. A final limitation we would like to address is that we assessed SCFs with EMA within a one-week period, whereas addictive behavior was assessed retrospectively over the previous year. Future studies should combine the EMA of SCFs and addictive behavior to better pool the data at the same time and data level.

## 5 Conclusion

Overall, we found evidence for a positive relationship between SCFs and addictive behavior at the between-person level, but no evidence for a predictive within-person relationship over time. Further exploratory analyses suggested several new hypotheses to be tested in future studies: (1) Addictive SCFs in daily life are early markers of an escalation of substance and non-substance related use and thus for addictive disorders. (2) An explicit monitoring of SCFs by apps may increase self-reflection and thereby promote the mobilization of cognitive control in response to goal-desire conflicts. In future studies, it will be important to use EMA to assess SCFs and addictive behavior at faster time scales to track fine-grained intraindividual fluctuations of self-control and addictive behaviors and assess associations between the two constructs on a day-to-day basis (Zech et al., 2022).

## Data availability statement

The datasets presented in this study can be found in online repositories. The names of the repository/repositories and accession number(s) can be found below: Open Science Framework (OSF) under https://osf.io/49e6d.

## Ethics statement

The studies involving humans were approved by the Institutional Review Board at the Technische Universität Dresden (IRB00001473). The studies were conducted in accordance with the local legislation and institutional requirements. The participants provided their written informed consent to participate in this study.

## Author contributions

AK: Conceptualization, Data curation, Formal analysis, Investigation, Methodology, Project administration, Visualization, Writing – original draft. MJ: Formal analysis, Methodology, Writing – review & editing. MW: Conceptualization, Investigation, Methodology, Project administration, Writing – review & editing. JF: Investigation, Project administration, Writing – review & editing. CB: Data curation, Validation, Writing – review & editing. K-MK: Conceptualization, Investigation, Methodology, Project administration, Writing – review & editing. GB: Conceptualization, Funding acquisition, Methodology, Resources, Supervision, Writing – review & editing. MS: Conceptualization, Funding acquisition, Methodology, Resources, Supervision, Writing – review & editing. TG: Conceptualization, Funding acquisition, Methodology, Resources, Supervision, Writing – review & editing.

## Footnotes

1
https://osf.io/gudys

2
https://osf.io/vmuyx

3
https://osf.io/49e6d
