# Supplementary material for: No evidence for a reciprocal relationship between daily self-control failures and addictive behavior in a longitudinal study
Source: Front Psychol. 2024 May 1;15:1382483. doi: 10.3389/fpsyg.2024.1382483 (PMC11095395; doi:10.3389/fpsyg.2024.1382483)
Supplement: Supplementary file 1 [file Data_Sheet_1.docx]

# Supplemental material

## Sample size calculation

The sample size for the whole project was estimated using Stata 13 for multiple linear regression with power (1- *β*) = .80, significance level *α*= 0.05, and five covariates (age, gender, IQ, income, school graduation). We assumed moderate group differences at baseline with R^2^=.05 according previous studies comparing individuals with gambling disorder, alcohol dependence, and Tourette syndrome on measures of executive functions and decision-making (Goudriaan et al. 2005; Goudriaan et al. 2006). The necessary sample size would have been *N* = 235 in total. Furthermore, we assumed a dropout rate of 30 % during the first funding phase of the project (3 years). The final estimated sample size was 330 with 110 in each group.

## Table S1

*Descriptive overview of the quantity of use, frequency of use and (modified) DSM-5 criteria of addictive disorders with median and range and separately for the assessment years*

|  | Quantity of use (see first column) | | | Frequency of use (days per week) | | | DSM-5 criteria (of the 11 criteria) | | |
| --- | --- | --- | --- | --- | --- | --- | --- | --- | --- |
|  | Baseline | 3-year FU | 6-year FU | Baseline | 3-year FU | 6-year FU | Baseline | 3-year FU | 6-year FU |
| n | 338 | 252 | 241 | 338 | 252 | 241 | 338 | 252 | 241 |
|  | *Median*  *(range)* | *Median*  *(range)* | *Median*  *(range)* | *Median*  *(range)* | *Median*  *(range)* | *Median*  *(range)* | *Median*  *(range)* | *Median*  *(range)* | *Median*  *(range)* |
| Tobacco  (cigarettes per day) | 0  (0-20) | 0  (0-30) | 0  (0-20) | 0  (0-7) | 0  (0-7) | 0  (0-7) | 0  (0-7) | 0  (0-6) | 0  (0-6) |
| Alcohol  (gram ethanol per occasion) | 36  (0-194) | 36  (0-158) | 27  (0-171) | 0.5  (0-7) | 2  (0-7) | 0.5  (0-7) | 0  (0-6) | 0  (0-6) | 0  (0-6) |
| Internet use (hours per occasion) | 1  (0-10) | 5  (0-5) | 5  (0-5) | 4  (0-7) | 0.5  (0-7) | 0.5  (0-7) | 0  (0-9) | 0  (0-7) | 0  (0-7) |
| Computer gaming  (hours per occasion) | 0  (0-5) | 0  (0-5) | 1  (0-10) | 0  (0-7) | 0.25  (0-7) | 0.25  (0-7) | 0  (0-8) | 0  (0-6) | 0  (0-4) |
| Gambling  (hours per occasion) | 0  (0-4) | 0  (0-1) | 0  (0-1) | 0  (0-2) | 0  (0-4) | 0  (0-2) | 0  (0-2) | 0  (0-2) | 0  (0-1) |
| Shopping  (hours per occasion) | 0  (0-3) | 0  (0-7) | 0  (0-4) | 0  (0-2) | 0  (0-2) | 0  (0-2) | 0  (0-1) | 0  (0-6) | 0  (0-4) |

## Table S2-1

*Descriptive statistics and correlations between self-control failures (SCF) and* ***quantity*** *of addictive use (Quant)*

|  | *M* | *SD* | SCF0 | SCF3 | SCF6 | Quant0 | Quant3 | Quant6 |
| --- | --- | --- | --- | --- | --- | --- | --- | --- |
| SCF0 | 0.53 | 0.26 | - |  |  |  |  |  |
| SCF3 | 0.53 | 0.27 | 0.27* | - |  |  |  |  |
| SCF6 | 0.55 | 0.28 | 0.30* | 0.36* | - |  |  |  |
| Quant0 | 0.37 | 0.26 | 0.12* | 0.21* | 0.29* | - |  |  |
| Quant3 | 0.74 | 0.28 | 0.04 | 0.15* | 0.03 | 0.41* | - |  |
| Quant6 | 0.60 | 0.39 | -0.04 | 0.08 | 0.10 | 0.22* | 0.36* | - |

Note: *M*=mean value; *SD*=standard deviation; ***** *p*<0.05

## Table S2-2

*Descriptive statistics and correlations between self-control failures (SCF) and* ***frequency*** *of addictive use (Frequ)*

|  | *M* | *SD* | SCF0 | SCF3 | SCF6 | Frequ0 | Frequ3 | Frequ6 |
| --- | --- | --- | --- | --- | --- | --- | --- | --- |
| SCF0 | 0.53 | 0.26 | - |  |  |  |  |  |
| SCF3 | 0.53 | 0.27 | 0.27* | - |  |  |  |  |
| SCF6 | 0.55 | 0.28 | 0.30* | 0.36* | - |  |  |  |
| Frequ0 | 6.71 | 4.44 | 0.12* | 0.15* | 0.32* | - |  |  |
| Frequ3 | 5.65 | 4.22 | 0.06 | 0.08 | 0.10 | 0.46* | - |  |
| Frequ6 | 4.04 | 4.11 | 0.05 | 0.12 | 0.18* | 0.24* | 0.51* | - |

Note: *M*=mean value; *SD*=standard deviation; ***** *p*<0.05

## Table S2-3

*Descriptive statistics and correlations between self-control failures (SCF) and* ***DSM-5 criteria*** *for addictive disorders (Sym)*

|  | *M* | *SD* | SCF0 | SCF3 | SCF6 | Sym0 | Sym3 | Sym6 |
| --- | --- | --- | --- | --- | --- | --- | --- | --- |
| SCF0 | 0.53 | 0.26 | - |  |  |  |  |  |
| SCF3 | 0.53 | 0.27 | 0.27* | - |  |  |  |  |
| SCF6 | 0.55 | 0.28 | 0.30* | 0.36* | - |  |  |  |
| Sym0 | 3.03 | 2.74 | 0.14* | 0.22* | 0.25* | - |  |  |
| Sym3 | 2.15 | 2.69 | 0.06 | 0.11 | 0.11 | 0.42* | - |  |
| Sym6 | 1.55 | 2.34 | 0.10 | 0.08 | 0.10 | 0.43* | 0.58* | - |

Note: *M*=mean value; *SD*=standard deviation; ***** *p*<0.05

## Table S3

*Descriptive statistics and correlations between self-control failures (SCF) and trait impulsivity measured with the Barratt Impulsivity Scale BIS11 (Imp)*

|  | *M* | *SD* | SCF0 | SCF3 | SCF6 | Imp0 | Imp3 | Imp6 |
| --- | --- | --- | --- | --- | --- | --- | --- | --- |
| SCF0 | 0.53 | 0.26 | - |  |  |  |  |  |
| SCF3 | 0.53 | 0.27 | 0.27* | - |  |  |  |  |
| SCF6 | 0.55 | 0.28 | 0.30* | 0.36* | - |  |  |  |
| Imp0 | 61.59 | 8.18 | 0.15* | 0.11 | 0.18* | - |  |  |
| Imp3 | 59.12 | 9.10 | 0.14* | 0.11 | 0.11 | 0.73* | - |  |
| Imp6 | 57.65 | 8.84 | 0.07 | 0.05 | 0.18* | 0.68* | 0.75* | - |

Note: *M*=mean value; *SD*=standard deviation; ***** *p*<0.05

## Table S4-1

*Parameter estimates for the random intercept cross‐lagged panel models (RI-CLPM; Hamaker et al., 2015) including self-control failures (SCFs) and* ***quantity of addictive behaviors***

|  |  |  |  | |  | | Confidence interval | |  | |
| --- | --- | --- | --- | --- | --- | --- | --- | --- | --- | --- |
| Predictor |  | Outcome | Unstandardized  coefficient | *p* | | Lower | | Upper | | Standardized coefficient |
|  | Autoregressive paths | |  |  | |  | |  | |  |
| SCFs 0 | → | SCFs 3 | -0.05 | 0.79 | | -0.39 | | 0.29 | | -0.05 |
| SCFs 3 | → | SCFs 6 | -0.05 | 0.79 | | -0.39 | | 0.29 | | -0.05 |
| Quantity 0 | → | Quantity 3 | 0.28 | 0.001 | | 0.12 | | 0.44 | | 0.25 |
| Quantity 3 | → | Quantity 6 | 0.28 | 0.001 | | 0.12 | | 0.44 | | 0.30 |
|  | Cross-lagged paths | |  |  | |  | |  | |  |
| SCFs 0 | → | Quantity 3 | -0.08 | 0.40 | | -0.25 | | 0.10 | | -0.06 |
| SCFs 3 | → | Quantity 6 | -0.08 | 0.40 | | -0.25 | | 0.10 | | -0.07 |
| Quantity 0 | → | SCFs 3 | -0.05 | 0.62 | | -0.26 | | 0.16 | | -0.06 |
| Quantity 3 | → | SCFs 6 | -0.05 | 0.62 | | -0.26 | | 0.16 | | -0.06 |
|  | Covariance (between) | |  |  | |  | |  | |  |
| Trait SCFs | ↔ | Trait Quantity | 0.01 | 0.02 | | 0.001 | | 0.02 | | 0.76 |

*Note.* Equality constraints have been added to autoregressive and cross-lagged paths. The model includes no covariates.

## Table S4-2

*Parameter estimates for the random intercept cross‐lagged panel models (RI-CLPM; Hamaker et al., 2015) including self-control failures (SCFs) and* ***frequency of addictive behaviors***

|  |  |  |  | |  | | Confidence interval | |  | |
| --- | --- | --- | --- | --- | --- | --- | --- | --- | --- | --- |
| Predictor |  | Outcome | Unstandardized  coefficient | *p* | | Lower | | Upper | | Standardized coefficient |
|  | Autoregressive paths | |  |  | |  | |  | |  |
| SCFs 0 | → | SCFs 3 | -0.04 | 0.83 | | -0.43 | | 0.34 | | -0.04 |
| SCFs 3 | → | SCFs 6 | -0.04 | 0.83 | | -0.43 | | 0.34 | | -0.04 |
| Frequency 0 | → | Frequency 3 | 0.01 | 0.82 | | -0.09 | | 0.11 | | -0.03 |
| Frequency 3 | → | Frequency 6 | 0.01 | 0.82 | | -0.09 | | 0.11 | | 0.01 |
|  | Cross-lagged paths | |  |  | |  | |  | |  |
| SCFs 0 | → | Frequency 3 | -0.14 | 0.25 | | -0.39 | | 0.10 | | -0.17 |
| SCFs 3 | → | Frequency 6 | -0.14 | 0.25 | | -0.39 | | 0.10 | | -0.09 |
| Frequency 0 | → | SCFs 3 | -0.03 | 0.63 | | -0.15 | | 0.09 | | -0.06 |
| Frequency 3 | → | SCFs 6 | -0.03 | 0.63 | | -0.15 | | 0.09 | | -0.02 |
|  | Covariance (between) | |  |  | |  | |  | |  |
| Trait SCFs | ↔ | Trait Frequency | 0.01 | 0.02 | | 0.002 | | 0.02 | | 0.37 |

*Note.* Equality constraints have been added to autoregressive and cross-lagged paths. The model includes no covariates.

## Table S4-3

*Parameter estimates for the random intercept cross‐lagged panel models (RI-CLPM; Hamaker et al., 2015) including self-control failures (SCFs) and* ***DSM-5 criteria of addictive disorders***

|  |  |  |  | |  | | Confidence interval | |  | |
| --- | --- | --- | --- | --- | --- | --- | --- | --- | --- | --- |
| Predictor |  | Outcome | Unstandardized  coefficient | *p* | | Lower | | Upper | | Standardized coefficient |
|  | Autoregressive paths | |  |  | |  | |  | |  |
| SCFs 0 | → | SCFs 3 | 0.03 | 0.87 | | -0.29 | | 0.34 | | 0.03 |
| SCFs 3 | → | SCFs 6 | 0.03 | 0.87 | | -0.29 | | 0.34 | | 0.03 |
| Criteria 0 | → | Criteria 3 | 0.22 | 0.20 | | -0.11 | | 0.54 | | 0.20 |
| Criteria 3 | → | Criteria 6 | 0.22 | 0.20 | | -0.11 | | 0.54 | | 0.27 |
|  | Cross-lagged paths | |  |  | |  | |  | |  |
| SCFs 0 | → | Criteria 3 | -1.67 | 0.048 | | -3.33 | | -0.01 | | -0.16 |
| SCFs 3 | → | Criteria 6 | -1.67 | 0.048 | | -3.33 | | -0.01 | | -0.21 |
| Criteria 0 | → | SCFs 3 | -0.01 | 0.49 | | -0.03 | | 0.02 | | -0.08 |
| Criteria 3 | → | SCFs 6 | -0.01 | 0.49 | | -0.03 | | 0.02 | | -0.08 |
|  | Covariance (between) | |  |  | |  | |  | |  |
| Trait SCFs | ↔ | Trait Criteria | 0.16 | 0.001 | | 0.04 | | 0.16 | | 0.61 |

*Note.* Equality constraints have been added to autoregressive and cross-lagged paths. The model includes no covariates.

## Table S5

*Parameter estimates for the* ***exploratory*** *random intercept cross‐lagged panel models (RI-CLPM; Hamaker et al., 2015) including* ***(potentially) addiction-related*** *self-control failures (SCFs) and* ***quantity of addictive behaviors***

|  |  |  |  |  | Confidence interval | |  |
| --- | --- | --- | --- | --- | --- | --- | --- |
| Predictor |  | Outcome | Unstandardized  coefficient | *p* | Lower | Upper | Standardized coefficient |
|  | Autoregressive paths | |  |  |  |  |  |
| SCFs 0 | → | SCFs 3 | 0.21 | 0.06 | -0.006 | 0.42 | 0.26 |
| SCFs 3 | → | SCFs 6 | 0.21 | 0.06 | -0.006 | 0.42 | 0.17 |
| Quantity 0 | → | Quantity 3 | 0.18 | 0.02 | 0.03 | 0.33 | 0.16 |
| Quantity 3 | → | Quantity 6 | 0.18 | 0.02 | 0.03 | 0.33 | 0.18 |
|  | Cross-lagged paths | |  |  |  |  |  |
| SCFs 0 | → | Quantity 3 | 0.12 | 0.01 | 0.03 | 0.21 | 0.15 |
| SCFs 3 | → | Quantity 6 | 0.12 | 0.01 | 0.03 | 0.21 | 0.12 |
| Quantity 0 | → | SCFs 3 | -0.06 | 0.57 | -0.25 | 0.14 | -0.05 |
| Quantity 3 | → | SCFs 6 | -0.06 | 0.57 | -0.25 | 0.14 | -0.05 |

*Note.* Equality constraints have been added to autoregressive and cross-lagged paths. The model includes the covariates age, gender, IQ, and group assignment at baseline. The model yielded a good model fit (*χ2* =13.602, *df* = 13, *p* = 0.402, RMSEA = 0.012 [0.000–0.056], CFI = 0.997, SRMR = 0.036).

## Table S6

*Parameter estimates for the* ***exploratory*** *random intercept cross‐lagged panel models (RI-CLPM; Hamaker et al., 2015) including* ***non-addictive*** *self-control failures (SCFs) and* ***quantity of addictive behaviors***

|  |  |  |  |  | Confidence interval | |  |
| --- | --- | --- | --- | --- | --- | --- | --- |
| Predictor |  | Outcome | Unstandardized  coefficient | *p* | Lower | Upper | Standardized coefficient |
|  | Autoregressive paths | |  |  |  |  |  |
| SCFs 0 | → | SCFs 3 | -0.16 | 0,35 | -0.49 | 0.17 | -0.16 |
| SCFs 3 | → | SCFs 6 | -0.16 | 0.35 | -0.49 | 0.17 | -0.14 |
| Quantity 0 | → | Quantity 3 | 0.23 | 0.01 | 0.07 | 0.38 | 0.2 |
| Quantity 3 | → | Quantity 6 | 0.23 | 0.01 | 0.07 | 0.38 | 0.22 |
|  | Cross-lagged paths | |  |  |  |  |  |
| SCFs 0 | → | Quantity 3 | -0.22 | 0.003 | -0.37 | -0.08 | -0.19 |
| SCFs 3 | → | Quantity 6 | -0.22 | 0.003 | -0.37 | -0.08 | -0.19 |
| Quantity 0 | → | SCFs 3 | -0.21 | 0.04 | -0.41 | -0.01 | -0.23 |
| Quantity 3 | → | SCFs 6 | -0.21 | 0.04 | -0.41 | -0.01 | -0.23 |
|  | Covariance (between) | |  |  |  |  |  |
| Trait SCFs | ↔ | Trait Quantity | 0.01 | 0.13 | -0.002 | 0.01 | 0.87 |

*Note.* Equality constraints have been added to autoregressive and cross-lagged paths. The model includes the covariates age, gender, IQ, and group assignment at baseline. The model yielded a good model fit (*χ2* =15.295, *df* =11, *p* = 0.169, RMSEA = 0.034 [0.000–0.071], CFI = 0.984, SRMR = 0.03).

## Table S7

*Parameter estimates for the* ***exploratory*** *random intercept cross‐lagged panel models (RI-CLPM; Hamaker et al., 2015) including* ***non-addictive*** *self-control failures (SCFs) and* ***frequency of addictive behaviors***

|  |  |  |  |  | Confidence interval | |  |
| --- | --- | --- | --- | --- | --- | --- | --- |
| Predictor |  | Outcome | Unstandardized  coefficient | p | Lower | Upper | Standardized coefficient |
|  | Autoregressive paths | |  |  |  |  |  |
| SCFs 0 | → | SCFs 3 | -0.14 | 0.42 | -0.48 | 0.2 | -0.14 |
| SCFs 3 | → | SCFs 6 | -0.14 | 0.42 | -0.48 | 0.2 | -0.13 |
| Frequency 0 | → | Frequency 3 | 0.04 | 0.63 | -0.11 | 0.18 | 0.07 |
| Frequency 3 | → | Frequency 6 | 0.04 | 0.63 | -0.11 | 0.18 | 0.02 |
|  | Cross-lagged paths | |  |  |  |  |  |
| SCFs 0 | → | Frequency 3 | -0.2 | 0.12 | -0.45 | 0.05 | -0.19 |
| SCFs 3 | → | Frequency 6 | -0.2 | 0.12 | -0.45 | 0.05 | -0.11 |
| Frequency 0 | → | SCFs 3 | -0.18 | 0.00 | -0.28 | -0.08 | -0.33 |
| Frequency 3 | → | SCFs 6 | -0.18 | 0.00 | -0.28 | -0.08 | -0.17 |
|  | Covariance (between) | |  |  |  |  |  |
| Trait SCFs | ↔ | Trait Frequency | 0.01 | 0.16 | -0.003 | 0.02 | 0.34 |

*Note.* Equality constraints have been added to autoregressive and cross-lagged paths. The model includes the covariates age, gender, IQ, and group assignment at baseline. The model yielded a good model fit (χ2 = 16.966, df =11, p = 0.109, RMSEA = 0.04 [0.000–0.076], CFI = 0.978, SRMR = 0.026).

## Table S8

*Parameter estimates for the* ***exploratory*** *random intercept cross‐lagged panel models (RI-CLPM; Hamaker et al., 2015) including* ***motivational*** *self-control failures (SCFs) (i.e. no attempt to resist a desire) and* ***frequency of addictive behaviors***

|  |  |  |  |  | Confidence interval | |  |
| --- | --- | --- | --- | --- | --- | --- | --- |
| Predictor |  | Outcome | Unstandardized  coefficient | *p* | Lower | Upper | Standardized coefficient |
|  | Autoregressive paths | |  |  |  |  |  |
| SCFs 0 | → | SCFs 3 | -0.27 | 0.07 | -0.56 | 0.02 | -0.3 |
| SCFs 3 | → | SCFs 6 | -0.27 | 0.07 | -0.56 | 0.02 | -0.2 |
| Frequency 0 | → | Frequency 3 | 0.02 | 0.78 | -0.13 | 0.17 | 0.04 |
| Frequency 3 | → | Frequency 6 | 0.02 | 0.78 | -0.13 | 0.17 | 0.01 |
|  | Cross-lagged paths | |  |  |  |  |  |
| SCFs 0 | → | Frequency 3 | -0.34 | 0.02 | -0.62 | -0.06 | -0.31 |
| SCFs 3 | → | Frequency 6 | -0.34 | 0.02 | -0.62 | -0.06 | -0.16 |
| Frequency 0 | → | SCFs 3 | -0.15 | 0.001 | -0.24 | -0.06 | -0.33 |
| Frequency 3 | → | SCFs 6 | -0.15 | 0.001 | -0.24 | -0.06 | -0.14 |
|  | Covariance (between) | |  |  |  |  |  |
| Trait SCFs | ↔ | Trait Frequency | 0.02 | 0.00 | 0.008 | 0.03 | 0.74 |

*Note.* Equality constraints have been added to autoregressive and cross-lagged paths. The model includes the covariates age, gender, IQ, and group assignment at baseline. The model yielded a good model fit (*χ2* =19.989, *df* =11, *p* = 0.046, RMSEA = 0.049 [0.007–0.083], CFI = 0.969, SRMR = 0.031).

## Table S9

*Parameter estimates for the* ***exploratory*** *random intercept cross‐lagged panel models (RI-CLPM; Hamaker et al., 2015) including* ***motivational*** *self-control failures (SCFs) (i.e. no attempt to resist a desire) and* ***DSM-5 criteria of addictive disorders***

|  |  |  |  |  | Confidence interval | |  |
| --- | --- | --- | --- | --- | --- | --- | --- |
| Predictor |  | Outcome | Unstandardized  coefficient | *p* | Lower | Upper | Standardized coefficient |
|  | Autoregressive paths | |  |  |  |  |  |
| SCFs 0 | → | SCFs 3 | -0.2 | 0.18 | -0.48 | 0.09 | -0.21 |
| SCFs 3 | → | SCFs 6 | -0.2 | 0.18 | -0.48 | 0.09 | -0.15 |
| Criteria 0 | → | Criteria 3 | 0.31 | 0.001 | 0.12 | 0.5 | 0.21 |
| Criteria 3 | → | Criteria 6 | 0.31 | 0.001 | 0.12 | 0.5 | 0.37 |
|  | Cross-lagged paths | |  |  |  |  |  |
| SCFs 0 | → | Criteria 3 | -2.03 | 0.03 | -3.82 | -0.25 | -0.16 |
| SCFs 3 | → | Criteria 6 | -2.03 | 0.03 | -3.82 | -0.25 | -0.17 |
| Criteria 0 | → | SCFs 3 | -0.02 | 0.14 | -0.04 | 0.01 | -0.16 |
| Criteria 3 | → | SCFs 6 | -0.02 | 0.14 | -0.04 | 0.01 | -0.18 |
|  | Covariance (between) | |  |  |  |  |  |
| Trait SCFs | ↔ | Trait Criteria | 0.07 | 0.01 | 0.02 | 0.12 | 0.41 |

*Note.* Equality constraints have been added to autoregressive and cross-lagged paths. The model includes the covariates age, gender, IQ, and group assignment at baseline. The model yielded a good model fit (*χ2* = 8.521, *df* =5 , *p* = 0.13, RMSEA = 0.046 [0.000–0.097], CFI = 0.991, SRMR = 0.023).

## Table S10

***Exploratory group difference tests*** *between motivational self-control failures (SCFs) (i.e. no attempt to resist a conflicting desire) and volitional SCFs (i.e. attempt to resist a conflicting desire) concerning reported conflict strength and desire types over all three time points.*

|  | Baseline | | | 3-year follow-up | | | 6-year follow-up | | |
| --- | --- | --- | --- | --- | --- | --- | --- | --- | --- |
|  | Motiva-  tional SCF | Volitional SCFs | Test value | Motiva-tional SCF | Volitional SCFs | Test value | Motiva-tional SCF | Volitional SCFs | Test value |
|  | *M (SD)* | *M (SD)* |  | *M (SD)* | *M (SD)* |  | *M (SD)* | *M (SD)* |  |
| Conflict strength | 2.9  (0.98) | 3.67 (0.99) | *t*(216) = 11.15; *p* = 0.00 | 2.78  (0.95) | 3.53  (1.06) | *t*(161) = 9.61; *p* = 0.00 | 2.80  (1.02) | 3.68 (0.95) | *t*(102) = 8.96; *p* = 0.00 |
|  |  |  |  |  |  |  |  |  |  |
| Desire type | Number | Number | Overall: *χ^2^*=173.21; *p* < 0.001 | Number | Number | Overall: *χ^2^*= 115.27; *p* < 0.001 | Number | Number | Overall: *χ^2^* = 70.59; *p* < 0.001 |
| Drinking alcohol | 46 | 12 | *χ^2^* = 11.14;  *p* = 0.001 | 48 | 8 | *χ^2^*= 19.966; *p*< 0.001 | 38 | 8 | *χ^2^*= 11.595; *p* = 0.001 |
| Smoking | 218 | 46 | *χ^2^* = 75.62;  *p*< 0.001 | 88 | 39 | *χ^2^* = 8.98;  *p*= 0.003 | 46 | 9 | *χ^2^* = 15.211; *p*< 0.001 |
| Gaming | 63 | 41 | *χ^2^* = 0.3;  *p*= 0.585 | 54 | 12 | *χ^2^*= 17.763; *p*< 0.001 | 35 | 8 | *χ^2^* = 9.747;  *p*= 0.002 |
| Watch-ing TV | 99 | 48 | *χ^2^*= 5.697;  *p* = 0.017 | 40 | 30 | *χ^2^* = 0.005;  *p*= 0.942 | 24 | 20 | *χ^2^*= 0.288;  *p* = 0.592 |
| Sleeping | 63 | 95 | *χ^2^* = 24.183; *p*< 0.001 | 46 | 76 | *χ^2^* = 19.691; *p*< 0.001 | 30 | 42 | *χ^2^*= 8.989;  *p*= 0.003 |
| Relaxing | 61 | 103 | *χ^2^* = 31.805; *p*< 0.001 | 51 | 69 | *χ^2^* = 10.817; *p* = 0.001 | 42 | 49 | *χ^2^* = 6.223;  *p*= 0.013 |
| Being unpolite | 35 | 41 | *χ^2^*= 4.634;  *p*= 0.031 | 18 | 42 | *χ^2^* = 18.237; *p* < 0.001 | 12 | 24 | *χ^2^* = 9.693;  *p*= 0.002 |
| Hygiene | 12 | 24 | *χ^2^* = 9.162;  *p* = 0.002 | 15 | 11 | *χ^2^*= 0.01;  *p*= 0.920 | 9 | 14 | *χ^2^* = 3.615;  *p*= 0.057 |

*Note.* M= mean; SD= standard deviation; We report only those desire types for which we found significant differences between motivational (i.e. no attempt to resist a desire) and volitional SCFS (i.e. attempt to resist a desire) at one of the three assessment waves.
